# Oxide Metallurgy Technology in High Strength Steel: A Review

**DOI:** 10.3390/ma15041350

**Published:** 2022-02-11

**Authors:** Wei Liang, Ruming Geng, Jianguo Zhi, Jing Li, Fei Huang

**Affiliations:** 1 State Key Laboratory of Advanced Metallurgy, University of Science and Technology Beijing, Beijing 100083, China; b20200579@xs.ustb.edu.cn (W.L.); ustbgrm@163.com (R.G.); huangfei19960923@163.com (F.H.); 2 Inner Mongolia Enterprise Key Laboratory of Rare Earth Steel Products Research and Development, Baotou 014010, China; 3 Inner Mongolia Baotou Steel Union Co., Ltd., Baotou 014010, China

**Keywords:** high strength steel, heat affected zone, oxide metallurgy technology, rare earth, rare earth inclusion, second phase

## Abstract

Oxide metallurgy technology plays an important role in inclusion control and is also applied to improve the weldability of high strength steel. Based on the requirements of the weldability in high strength steel, the influencing factors of weld heat affected zone (HAZ) as well as the development and application status of oxide metallurgy technology are summarized in this review. Moreover, the advantages and difficulties in the application of rare earth (RE) oxide metallurgy technology are analyzed, combined with the performance mechanism of RE and its formation characteristics of fine and high melting point RE inclusions with distribution dispersed in liquid steel. With the weldability diversities of different high strength steels, the research status of weldability of high strength steel with high carbon equivalent and the effects of RE on the microstructure and properties of HAZ are discussed, and some suggestions about further research in the future are proposed.

## 1. Introduction

High strength low alloy (HSLA) steel, a weldable low carbon engineering structure steel, is obtained by adding a small amount of Mn, Si, Nb, V, Ti, Al and other alloy elements into ordinary carbon steel and subjecting it to a heat treatment process. Because of low C content, low crack sensitivity, fine microstructure, and high strength and toughness, it is widely used in bridges, buildings, ships, high pressure vessels, construction machinery, and other fields [1]. Furthermore, the comprehensive properties of high strength steel have been improved significantly with the development and application of refining technology, thermo mechanical control process (TMCP), thermomechanical treatment, and thin slab continuous casting and rolling [2,3]. It is well known that welding is the most common means of building engineering structures. However, the toughness of the weld HAZ is significantly lower than that of base metal after the welding thermal cycle in high strength steel, possibly resulting in structural failure. In order to avoid significant deterioration of HAZ toughness, in recent years, fundamental measures have been adopted to improve the weldability of the base metal matrix by determining the influencing factors of microstructure and properties of HAZ, in addition to using the appropriate welding process. A series of oxide metallurgy technologies have been developed [4,5] that control the formation of fine and dispersed high melting point inclusions in liquid steel, and they affect the size, morphology, and distribution of sulfide, carbonitride, and other non-metallic inclusions. The technologies have improved the weldability of high strength steel by making full use of these inclusions to pin the austenite grain boundary and induce the formation of acicular ferrite (AF) during the welding thermal cycle [6].

As an “industrial vitamin”, there are many studies on the function mechanisms of rare earth (RE) in steel. The studies using RE to modify inclusions and improving steel microstructure and properties through the control of RE inclusions are also gradually increasing [7,8,9]. In addition, disperse high melting point RE inclusions are used to pin the austenite grain boundary and induce AF nucleation during the welding, which can also play the role in oxide metallurgy technology [10]. However, for high strength steel with high carbon equivalent, it is difficult to form AF, and easy to produce brittle structures such as upper bainite. There is great importance to study the measures to improve the weldability and the function mechanisms of RE in HAZ.

Based on the control of microstructure and properties of HAZ in high strength steel, this paper assesses the influencing factors of microstructure and properties in HAZ and the development and application of oxide metallurgy technology. Moreover, the effects of RE oxide metallurgy technology on HAZ are also presented. For the weldability of high strength steel with high carbon equivalent, the current measures and the influences of RE on improving the microstructure and properties of HAZ are discussed. The theoretical supports for enriching the application mechanism of RE in steel and broadening oxide metallurgy technology are further provided.

## 2. Research Status of Oxide Metallurgy Technology in Steel

### 2.1. Influencing Factors of Microstructure and Properties in HAZ

**Figure 1:**
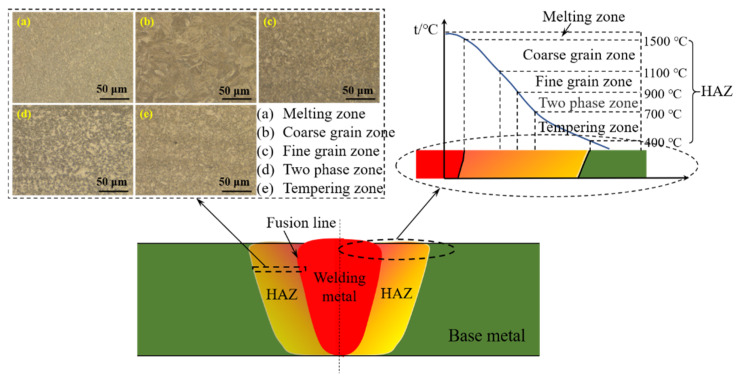
Structure and temperature range of HAZ in high strength steel. (Reproduced with permission from ref. [13]. Copyright 2008 China Academic Journal Electronic Publications.)

The welding HAZ is a whole zone where the structure and properties of the unmelted base metal on both sides of the weld fusion line change under the conditions of the thermal cycle. The microstructure and temperature range of HAZ in high strength steel are shown in Figure 1. According to the distance from the weld center, it is divided into a melting zone, coarse grain zone, fine grain zone, two phase zone, and tempering zone, and the corresponding microstructure and temperature range are also shown. The coarse grain zone has large grains and poor toughness, so it is the weak zone of the HAZ and of entire welded joint. In addition, chemical composition, austenite grain size, and cooling speed are identified as the major factors that affect the microstructure and properties of HAZ [11,12].

#### 2.1.1. Chemical Composition

**Figure 2:**
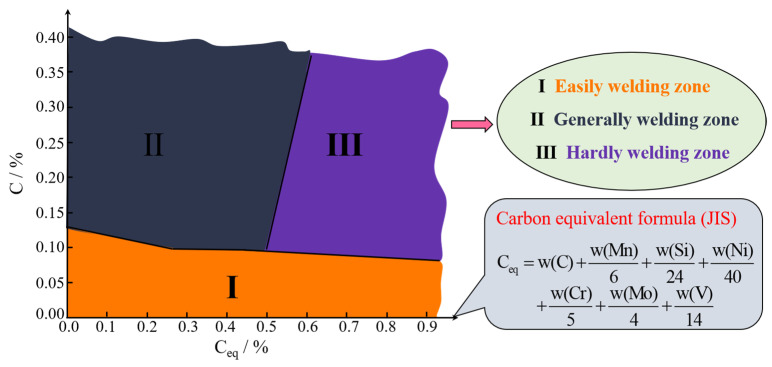
Effects of carbon content and carbon equivalent on weldability of high strength steel. (Reproduced with permission from [23]. Copyright 2004 China Academic Journal Electronic Publications.)

In high strength steel, the increased C content can reduce the ductility and toughness and improve the sensitivity of the welding crack. As for the steel with good weldability, the C content is generally not more than 0.20 wt%, or even lower. The effects of carbon content and carbon equivalent on the weldability of steel are described in Figure 2. Zone I is the easy welding zone (around C < 0.13%, Ceq > 0), and the steel in this zone can be welded directly. Zone II is the general welding zone (around C > 0.13%, 0 < Ceq < 0.5%); the steel in this zone has high requirements for the welding process, so preheating before welding and heat treatment after welding are ordinarily required to ensure the performance of welded joints. Zone III is the hard welding zone (around C > 0.1%, Ceq > 0.5%), and the steel in this zone has low requirements for weldability. Mn is usually added to steel to offset the strength loss due to carbon reduction. The initial transformation temperature of ferrite is decreased by adding Mn, which promotes the formation of AF and affects the toughness of the HAZ [14]. In contrast, the Si content increased in steel can inhibit the formation of AF and increase content of the grain boundary ferrite (GBF) and M-A structure [15], and it leads to lower ductility and toughness. The content of S and P is generally limited to a low range, otherwise the weldability of the steel will deteriorate. Al is a strong deoxidizer but also a nitrogen fixing agent. The austenite grain boundary can be effectively pinned by nanoscale AlN below 1100 °C, whereas the composite inclusion of Al also induces AF nucleation and refines grains [16]. However, Al_2_O_3_ inclusions aggregate easily to form clusters, becoming a crack source. With proper content of Nb, Ti and C, N in steel, fine carbonitrides can be easily formed, which together play the role of pinning grain boundaries in the welding thermal cycle [17]. Oxides and nitrides of Ti used to induce AF also improve welding toughness [18]. Nevertheless, the excessive Ti and Nb will lead to coarsening carbonitrides and deteriorating toughness. The carbonitrides of V precipitated in the austenite zone can inhibit the grain growth of austenite, and carbonitrides precipitated in the ferrite zone can increase the nucleation core of AF [19]. However, when welding heat input is high, they will not work. The formation temperature of carbides containing Cr is low, such as M_7_C_3,_ which basically cannot play a role in the welding thermal cycle. Ni is an element that expands the austenite zone. It can affect the diffusion rate of carbon and alloy elements, improve the hardenability, and slow down the hardening cracking trend during welding [20]. Generally, proper Mo in steel enables complete bainite structure to be obtained in a wide range of cooling speeds and realize the uniformity of HAZ structure [21]. The segregation of trace B at the grain boundary can reduce the grain boundary energy so as to decrease the driving force of grain growth during welding [22]. Moreover, BN also promotes ferrite nucleation during cooling. Therefore, the content of carbon and other alloys should be considered in steel design, and the characteristics of disperse inclusion particles should be also fully utilized.

#### 2.1.2. Austenite Grain Size

**Figure 3:**
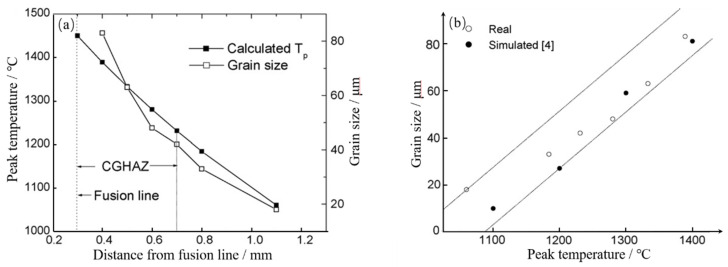
Peak temperature and austenite grain size of HAZ in HSLA-100 steel. (**a**) The relationship of peak temperature and distance from fusion line; (**b**) the relationship of grain size and peak temperature. (Reproduced with permission from [24]. Copyright 2007 Elsevier Publications.)

The toughness of the HAZ in steel is primarily affected by austenite grain coarsening, and its coarsening is closely related to welding heat input. Shome [24] studied the relationship between the peak temperature and austenite grain size of HAZ in HSLA-100 steel. The peak temperature of fusion line in HAZ is 1450 °C, and the peak temperature and grain size decrease with the increasing distance from the fusion line, as shown in Figure 3a. With the increase of peak temperature, the grain size also increases significantly, as shown in Figure 3b.

Further, the grain size is also affected by the second phase particles. Most of the particles begin to dissolve at 1200 °C with grain coarsening. The coarsening of grain has a direct influence on its transformation in the cooling process and the morphology of transformation products, thus affecting the weldability of steel to some degree. The relationship between austenite grain size (D), second phase size (d), and second phase volume fraction (f) is expressed by the follow equation [25]:

(1) $$D=\frac{\pi d}{6f}\left(\frac{3}{2}-\frac{2}{Z}\right)$$

where Z is used to represent the ratio of the maximum grain diameter to the average grain diameter. It is concluded that the size and volume fraction of second phase precipitates is of great importance to grain growth. Therefore, the relation of welding heat input and second phase should be balanced according to the service conditions of different types of steel in order to form the optimal grain size.

#### 2.1.3. Cooling Speed

In addition to austenite grain coarsening, cooling speed also affects the microstructure and properties of HAZ in steel. The cooling speed after welding is expressed by the cooling time (t_8/5_) between 800 °C and 500 °C. Generally, the transformation microstructures are martensite and bainite when t_8/5_ is short. The microstructures are primarily GBF, ferrite side plate (FSP), and AF when t_8/5_ is medium. They are primarily polygonal ferrite (PF), GBF, and pearlite when t_8/5_ is long [26,27]. Fang et al. [28] studied the microstructure evolution of the welding coarse grain zone in V-Ti steel. The results showed that when t_8/5_ was 7.5 s, the coarse grain zone was primarily martensite lath. GBF, PF, and some granular bainite were formed with t_8/5_ changing from 20 s, to 40 s, to 100 s, and the ferrite size increased significantly.

**Figure 4:**
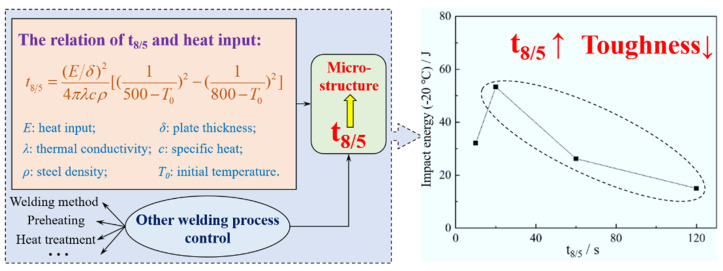
Influence factors of t_8/5_ and its relationship with low temperature impact toughness of HAZ. (Reproduced with permission from [29]. Copyright 2013 Elsevier Publications.)

The influencing factors of t_8/5_ and its relationship with low temperature impact toughness of HAZ are shown in Figure 4. When affecting the microstructure of HAZ, t_8/5_ is directly related to heat input, plate thickness, thermal conductivity, specific heat, steel density, and initial temperature, and indirectly related to welding methods, preheating, and heat treatment after welding. Within limits, the impact toughness of HAZ decreases significantly when t_8/5_ is increased, as shown in Figure 4. More importantly, heat input, welding mode, preheating (affecting initial temperature), and heat treatment after welding are controllable among the relevant factors of t_8/5_ and these factors should be the subjects of future research focused on obtaining excellent microstructures.

### 2.2. Development of Oxide Metallurgy Technology

**Figure 5:**
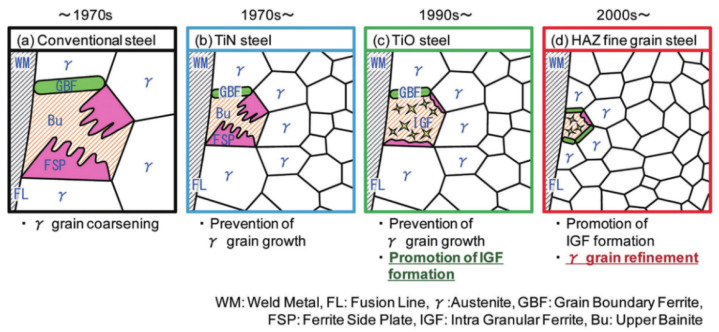
Development of HAZ toughening technology at Nippon Steel. (Reproduced with permission from [32]. Copyright 2018 Nippon Steel Corporation Publications.)

Many studies and practical work were initially carried out by Japanese scholars on the application of oxide metallurgy technology, which effectively improves the weldability of high strength steel. Nippon Steel has long been committed to utilizing oxide metallurgy technology to improve the HAZ toughness of steel, and its development process is shown in Figure 5. In the 1970s, fine and dispersed TiN particles were proposed to inhibit austenite grain coarsening during welding so as to reduce the brittleness of HAZ. Subsequent studies found that TiN dissolved or aggregated easily when the peak welding temperature exceeded 1400 °C. The technology of Ti deoxidation treatment had been used to form stable high melting point oxide inclusions in steel since the 1990s [30]. And Ti_2_O_3_ inclusions and composite inclusions could be used to promote AF nucleation. However, Ti_2_O_3_ aggregated and grew easily in refined liquid steel, and it was difficult to obtain fine inclusions. After the year of 2000, super high HAZ toughness technology with fine microstructure impacted by fine particles (HUTFF) as the third-generation oxide metallurgy technology was developed. It was based on the characteristic that basic metal (such as Mg, Ca) oxides or sulfides could stably exist at 1400 °C. These inclusions were to pin austenite grain boundaries and induce AF during cooling [31], which refined microstructure and improved welding toughness. Nippon Steel then increased the ability to induce AF nucleation by adjusting the alloy compositions to promote the formation of Mn-depleted zone according to HUTFF technology [32].

In 2004, JFE Shoji Trade Corporation (JFE) successfully developed excellent quality in large heat input welded joints (EWEI) technology to improve the toughness of HAZ from three aspects: controlling the grain size, adjusting the intragranular structure, and optimizing the alloy composition and production process [33]. The specific processes included raising the TiN solution temperature, reducing carbon equivalent, and increasing the nucleation particles of BN and Ca inclusions, and using a super online accelerated cooling (super-OLAC) process, respectively [34,35,36]. In addition, the formation of Ti oxide and the segregation of B at the grain boundary in steel were controlled by Kobe Steel through adjusting the content of Ti and B in the 1980s, which worked together on the pinning grain boundary and the nucleation of AF. Kobe super toughness (KST) technology was expanded to improve welding toughness [37,38]. Subsequently, the composition was adjusted around the fine control of the bainite structure of high strength steel to improve the driving force of phase transformation, and “low-carbon multi-directional bainite” composition technology was developed [39].

In addition, Chinese enterprises and scientific research institutes have also done relevant research on oxide metallurgy technology. The researchers of Wuhan Iron and Steel Group Corp. (WISCO) strictly controlled the content of carbon and alloy when designing the steel composition. Dispersed micron Ti, Nb carbonitrides and nanoscale high melting point Mg, Ca oxysulfide were used to pin high temperature austenite grain boundaries and induce AF. Thus, steel grades with heat input of 50–200 kJ/cm and 200–400 kJ/cm were developed, respectively [40,41]. Baosteel researchers conducted studies on revealing the metallurgical mechanism of Mg, Ca oxides. The fine and dispersed oxide sulfide particles generated in the steel were fully utilized to pin the grain boundary and induce AF effectively. High strength steel with excellent weldability was developed: the average impact energy at −20 °C was 142 J under 400 kJ/cm heat input [42]. With the production of low-carbon microalloyed steel, the researchers of Anshan Iron and Steel Group Corporation (AISC) adopted an unconventional deoxidation alloying process to control the formation of Ti composite inclusions with the size of 1 μm, inducing and refining ferrite grains. Compared with the conventional deoxidation process, the tensile strength and low temperature impact strength of the steel were increased by 24.56% and 24.00%, respectively. A range of ship plate steel products for high heat input welding were developed [43]. Furthermore, Yang’s team from Shanghai University had long studied the effects of Mg treatment on inclusions in steel and the properties of welding HAZ, and they concluded that Mg treatment could refine inclusions and improve the toughness of HAZ [44,45,46]. Scholars from Northeastern University found that the low temperature impact toughness of HAZ were optimum when the Zr content in low carbon steel was 0.003%. The 0.5~3 μm composite inclusion formed with the appropriate addition of Zr in steel could induce AF nucleation and refine grains during welding thermal simulation cooling [47]. The effects of different composite deoxidizers (V-Ti, Ti-Al (or RE), Ti-Ca, etc.) on the microstructure and properties of HAZ were also studied [48,49,50]. Among them, Xia from Central Iron and Steel Research Institute (CISRI) added trace Mg and Zr to the Ti-treated steel. The size of Ti composite inclusions was reduced significantly, which promoted AF nucleation and improved the toughness of HAZ. The developed E36 high heat input ship plate steel could achieve heat input of 240 kJ/cm [51]. Similarly, scholars from North China University of Science and Technology used Mg-Al-Ti compound deoxidation to produce 40 mm thick ship plate steel; the impact energy near the fusion line could reach 80% of the base metal under 150 kJ/cm heat input [52].

Currently, oxide metallurgy technology used to improve the properties of HAZ in high strength steel has been partially industrialized. There are still some problems, however, such as accurately controlling the content of Ti, Ca, Mg, and Zr, and aggregating and easily growing high melting point inclusions. Therefore, the additional methods of alloy elements, refining processes, and fine dispersion control technology of inclusions should be further optimized, and the types of beneficial inclusions and their nucleation effect on AF also need to be clarified. With the development and application of RE resources, the studies on its function mechanisms in steel have made some progress. Results of previous research indicated that trace RE added to high strength steel may significantly improve the toughness of HAZ [53].

## 3. Research Status of RE Oxide Metallurgy Technology in Steel

### 3.1. Development of RE Oxide Metallurgy Technology

Generally, the functions of RE in steel are purifying liquid steel, improving inclusions, and microalloying. As the most abundant RE elements in minerals, Ce and La are the most widely used in iron and steel metallurgy industry because of their active chemical properties and low price. Trace RE added to clean steel can remove O and S but can also reduce the segregation of harmful elements at grain boundaries through location competition and interaction [54]. The expansion coefficient and elastic modulus of RE inclusions are similar to those of the matrix, and its shapes are mostly circular and elliptical, which decrease additional stress and increase crack propagation energy during hot deformation and cooling [55]. In addition, the segregation of RE atoms affects the diffusion of other elements and the nucleation and growth of new phases, thus realizing microalloying [56].

Many high melting point oxides and sulfides can be formed in liquid steel because of the strong binding force between O, S, and RE. With the decrease of solubility product, new RE inclusions will continue to form during solidification, which achieves the requirements of oxide metallurgy technology for small size and dispersion of beneficial inclusions to some degree and promotes the development of RE oxide metallurgy technology.

**Figure 6:**
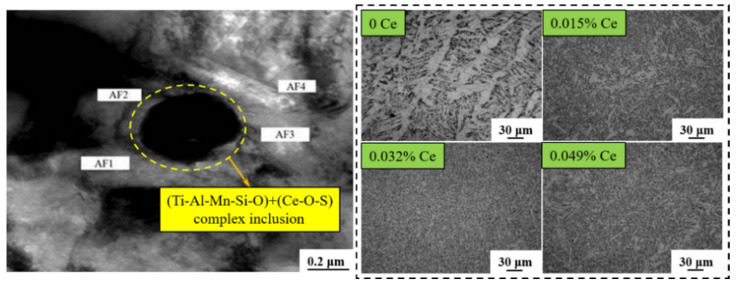
Microstructure of weld metal with different RE content. (Reproduced with permission from [57]. Copyright 2012 China Academic Journal Electronic Publications.)

Through welding E36 steel with RE welding wire, Yu et al. [57] found that the inclusions in the weld metal were the composite inclusions of (Ti-Al-Mn-Si-O) + (Ce-O-S) with the diameter of 0.5 μm, and the AF was induced by them, as shown in Figure 6. In addition, the areas of AF in the weld metal were also increasing with added RE content, which could reach 85% when the Ce content was 0.032%, and the impact energy at −40 °C exceeded 82 J. Deng et al. [58] studied the evolution of inclusions in steel and the nucleation mechanism of AF through experiments and thermodynamic calculations. It was concluded that (Mn-Al-Si-Ti-La-Ce-O) + MnS composite inclusions could be used as the particle of AF nucleation. The studies of Thewlis and Xue et al. [59,60] also indicated that the morphology and distribution of inclusions were improved by RE, and RE inclusions could induce AF nucleation easily, refine the microstructure of weld metal, and improve the weldability of steel. Moreover, Cao et al. [10] observed that the disperse RE inclusions in HAZ pinned the grain boundary and were beneficial to grain refinement after adding Ce into X70 steel. Some research studies of RE oxide metallurgical technologies are shown in Table 1.

**Figure 7:**
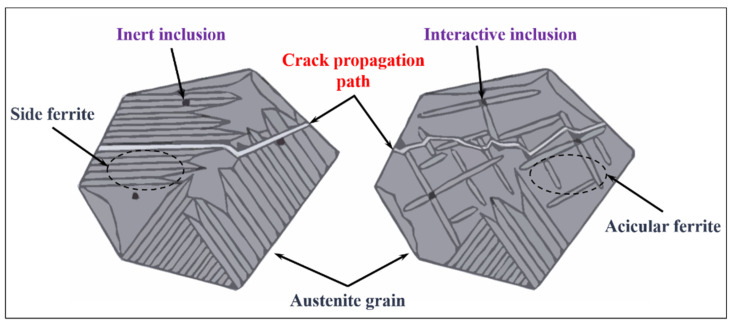
Schematic diagram of crack propagation path between FSP and AF. (Reproduced with permission from [66]. Copyright 2016 Doctoral Electronic Journal Publications.)

The propagation path of cleavage crack of AF nucleated by RE inclusions is different from that of FSP. As shown in Figure 7, the FSP has the sheet structure parallel to the crystal orientation, which can provide a better path for crack propagation and reduce the crack propagation energy. However, the structure of AF lath crosses each other, which has a strong effect of hindering crack propagation [66]. Consequently, the fracture toughness of steel is improved with the increase of AF.

At present, the formation mechanisms of AF mainly include the solute element change mechanism, low mismatch mechanism, stress-strain mechanism, and inert interface energy mechanism. The solute element change mechanism refers to the absorption of austenitic stabilization elements (C, Mn, B, etc.) by non-metallic inclusions, resulting in the decrease of solute element content on the surface of inclusions, the advance of austenite to ferrite transformation, and the increase of AF nucleation probability [12]. The low mismatch mechanism means studying the lattice structure matching degree between non-metallic inclusions and α-Fe, and it is conducive to the reduction of interface energy and stress energy required for ferrite nucleation with a high matching degree [67]. For example, the mismatches between Ce_2_O_3_, Ce_2_O_2_S, CeAlO_3_, and α-Fe are only 4.1%, 1.2%, and 7.0%, respectively, so that they all have the chance to effectively induce the particles of AF [68]. Thus, the possibility of ferrite nucleation induced by inclusions can be explained theoretically by calculating the mismatches between different inclusions and α-Fe. The two mechanisms noted are highly thought to explain the AF induction mechanism, yet there are still cases that cannot be fully explained by a single mechanism. Koseki [69] studied the influencing factors of AF in the weld metal and HAZ, and considered that the nucleation mechanism of AF in the weld metal was mainly the low mismatch mechanism, whereas the solute element change mechanism was mainly in HAZ. Furthermore, the combined action of two or more mechanisms should be discussed for the inclusions with different morphology and distribution.

### 3.2. Some Application Difficulties of RE Oxide Metallurgy Technology

Most scholars are focused on the study of the modification process of inclusions after RE addition and the capability of AF induced by different RE inclusions, yet the key technologies of controlling fine and dispersed RE inclusions are rarely studied [70,71,72]. As is generally known, RE in solid solution and RE inclusions are the main forms of RE in steel. The stability of total RE and different forms of RE in steel is crucial to ensuring the stability of RE action. The implementation of the latter depends on the stability of liquid steel cleanliness before RE addition, which is also conducive to the stable control of RE yield. The content of T.O, S, and RE have a significant influence on the formation and transformation of inclusions in RE-treated steel, thus the effects of T.O and S content on the quantity, type, size, and precipitation temperature of RE inclusions, as well as the cooperative control range of Ce, T.O, and S should be further analyzed.

**Figure 8:**
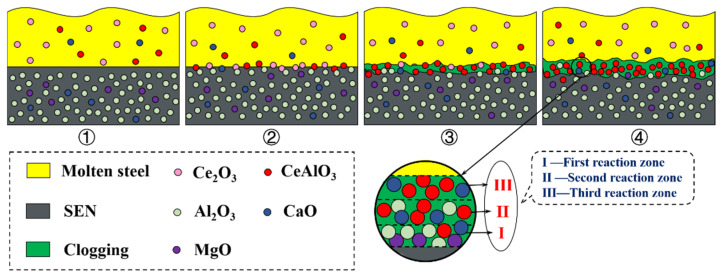
Schematic diagram of clogging formation in SEN. ① Inclusion suspension, ② Inclusion reaction, ③ Clogging formation, ④ Clogging aggregation (Reproduced with permission from [73]. Copyright 2021 Springer Nature Publications.)

**Figure 9:**
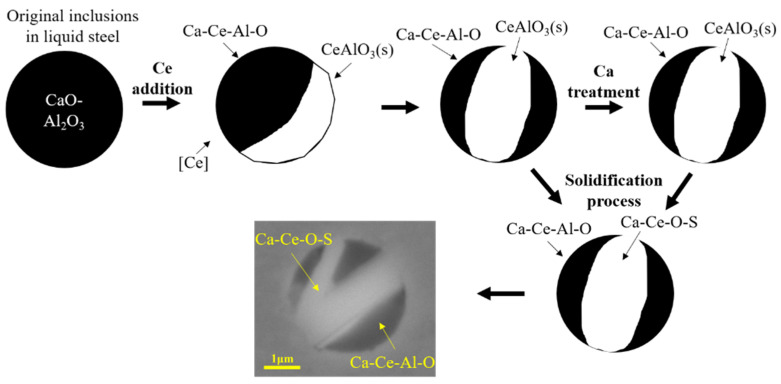
Evolution mechanism of inclusions in refining process. (Reproduced with permission from [74]. Copyright 2021 Iron and Steel Institute of Japan Publications.)

In addition, key trace elements such as Ca and Mg also need to be systematically studied for the transformation of inclusions and the reaction with refractory in the refining process. In the case of carrying out RE treatment and cancelling Ca treatment, the authors’ previous study indicated that the high melting point inclusion Ce_2_O_3_ formed in liquid steel reacted with Al_2_O_3_ in the refractory of the submerged entry nozzle (SEN) to form CeAlO_3_ [73]. The reaction products and other inclusions continued to accumulate, resulting in the formation of a clogging layer on the nozzle surface, as shown in Figure 8. By carrying out RE treatment and then Ca treatment in refining, Geng et al. [74] found that composite inclusions of Ca, Al, and RE were formed, as shown in Figure 9, which may not be beneficial to the oxide metallurgy of RE inclusions. Therefore, the effects of only RE treatment, the sequence of Ca treatment and RE treatment, and the addition amount of them on inclusion evolution, SEN clogging, and plate properties should be studied, so the optimization of refining process and the application of RE oxide metallurgy technology can be improved.

The relevant studies on RE oxide metallurgy technology have been primarily carried out under laboratory conditions, and the addition amount of RE is more than 150 ppm for the mold casting process used. In the actual process of continuous casting, the contact reactions between RE and air, steel slag, and refractory may cause problems such as large RE burning loss, low yield, erosion of refractory, SEN clogging, and so on [75]. How to achieve RE oxide metallurgy and stabilization control of RE under the condition of ensuring continuous casting is the key to restricting the industrial application of RE. The addition time, amount and homogenization control of RE, and the high density of RE deoxidation products that are difficult to remove are also problems that need to be addressed at present. Therefore, corresponding studies need to be developed from the aspects of purity and addition method of RE alloy, the compositions of refractory, stirring, and flow field distribution of liquid steel.

The carbon equivalents of most experimental steel products are less than 0.4% in previous research. The AF in HAZ is induced through controlling the formation of RE inclusions, and the welding toughness can be improved accordingly. For high strength steel with high carbon equivalent, however, the brittle structures such as upper bainite and granular bainite (consisting of ferrite and M-A structure) are easily formed in HAZ, whereas AF is difficult to form, leading to the traditional idea that oxide metallurgy cannot be applied. Thus, the weldability of high strength steel with high carbon equivalent and the effects of RE on the microstructure and properties of its HAZ must be explored further.

## 4. Research on Improving Weldability of High Strength Steel with High Carbon Equivalent

### 4.1. Research Status of Weldability in High Strength Steel with High Carbon Equivalent

**Figure 10:**
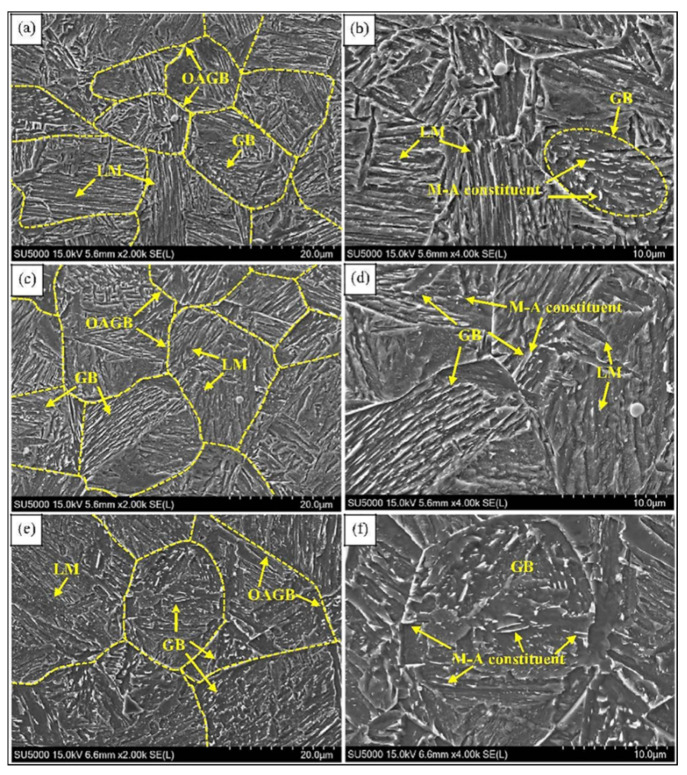
Microstructure of HAZ in high strength steel: (**a**,**b**) 3.9 kJ/cm; (**c**,**d**) 5.2 kJ/cm; (**e**,**f**) 7.75 kJ/cm. (Reproduced with permission from [78]. Copyright 2017 Elsevier Publications.)

The carbon equivalent of high strength steel with high carbon equivalent is usually more than 0.4%. This is located in Zone II (Figure 2) due to the high carbon content and carbon equivalent. The microstructure of HAZ in this type of steel is different from that of high strength steel with low carbon equivalent (ferrite, pearlite, etc.), so the mechanism of toughness deterioration is also different. Guo et al. [76] studied the microstructure and properties of welded joints in Q690C low carbon bainite steel. The results indicated that large parallel upper bainite lath bundles and M-A structure were formed in the welding fusion zone and coarse grain zone, which caused the hardening of HAZ and affected the impact toughness of welded joints. By adopting the methods of simulated welding, Shi et al. [77] studied the effect of cooling speed on HAZ of 800 MPa high strength steel. It was concluded that the austenite grain size increased with t_8/5_ extended; furthermore, the volume fraction of martensite decreased, the quantity of granular bainite increased, and the fracture toughness of HAZ decreased. Wang et al. [78] studied the microstructure changes of HAZ in Nb-Ti-Mo microalloyed steel with hot rolled 8 mm thick under different welding heat input and found that the austenite grain size increased significantly with the heat input raised. In addition, the fraction of martensite lath decreased and the fraction of granular bainite increased, as shown in Figure 10. The results proved that the coarsening of austenite grain and the formation of upper bainite and M-A structure in HAZ of high strength steel with high carbon equivalent are the important factors affecting weldability.

The previous studies on the weldability of this type of high strength steel mostly focus on the welding field, such as welding methods, welding wire composition and properties, preheating and heat treatment after welding, and heat input. The purposes are to obtain the structure with excellent comprehensive performance in the welding cooling. When studying the welding process of 900 MPa high strength steel, Gao et al. [79] adopted the methods of setting the preheating temperature above 125 °C, controlling the heat input with 1.04 kJ/cm, and adopting the temperature between weld beads to 150 °C to improve the tensile and impact properties of welded joints and prevent the generation of cold cracks. Xu [80] welded Q890/Q960 shaped steel with three different welding wires; the results indicated that the root crack rates of the welded joint with the three welding wires were less than 20% with preheating temperature of 100~150 °C and the heat input of 12~20 kJ/cm. Although the measures noted are beneficial to improving the welding toughness and reducing the cold crack, they will inevitably increase the welding cost and decrease the production efficiency. Therefore, the studies on improving the weldability of the base metal should be continued. By combining the function mechanisms in steel, RE can be fully used to improve the microstructure and welding toughness of HAZ.

### 4.2. Effect of RE on Weldability in High Strength Steel with High Carbon Equivalent

According to microalloying, RE in solid solution affects the microstructure transformation and grain size in steel. Through observing the continuous cooling transformation (CCT) curve and microstructure of RE containing steel, Liu et al. [81] found that RE made the entire CCT curve change downward to the right, the stability of undercooled austenite was increased, and the precipitation of proeutectoid ferrite and the transformation of bainite were delayed. The segregation of RE on grain boundaries and dislocation lines not only blocks carbon diffusion and carbide precipitation, affects the distribution of carbide among ferrite lath, and delays the formation of feathery upper bainite [82], but also decreases stacking fault energy of austenite, weakens martensite nucleation, and refines martensite lath [83].

Wang et al. [84] studied the influence of different Ce content on the transformation and morphology of pearlite in heavy rail steel, and it was concluded that RE could significantly inhibit the growth of austenite grain, as shown in Table 2. Relevant studies also indicated that the enrichment of RE at the grain boundary decreased the grain boundary energy and surface tension of austenite, reduced the driving force of grain growth, and promoted grain refinement [85]. Moreover, RE could homogenize the austenite grain size and increase the proportion of large angle grain boundaries, hindering the formation and propagation of cracks and effectively improving the strength and toughness of steel [86].

**Figure 11:**
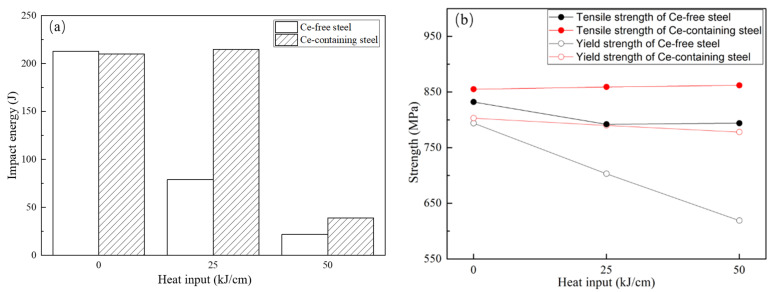
Mechanical properties of HAZ in Ce-free and Ce-containing steel: (**a**) effect of heat input on impact energy; (**b**) effect of heat input on strength. (Reproduced with permission from [92]. Copyright 2021 Taylor & Francis Publications.)

Previous studies have shown that it is feasible to apply RE and RE inclusions to affect the microstructure and grain of high strength steel with high carbon equivalent, improving the weldability of steel [87,88]. Lu et al. [89] studied the effects of Ce on the microstructure and toughness of HAZ in 700 MPa high strength steel. The results indicated that Ce delayed the bainite transformation, and the fine RE inclusions pinned the austenite grain boundary and increased the toughness. Similar results were obtained in subsequent research; Lu believed that RE improved the welding toughness of steel [90,91]. Geng et al. [92] analyzed the influence of Ce on the precipitation of inclusions in 700 MPa high strength steel and the properties of HAZ and found that RE and RE inclusions in Ce containing steel could also pin austenite grain boundaries, improve the crack propagation resistance of HAZ, and increase the mechanical properties, as shown in Figure 11. Therefore, the characteristics that RE and RE inclusions affecting the upper bainite transformation, refining the martensite lath and the austenite grain, and pinning the grain boundary can be used to reduce the brittle structure of HAZ.

Furthermore, microalloys such as Ti, Nb, Cr, and V are usually added into high strength steels. Many fine and dispersed Nb, Ti carbonitrides precipitate during hot deformation and heat treatment, which significantly improves the strength and toughness of the base metal due to grain refining and precipitation strengthening. The second phase (Nb, Ti carbonitrides) can also improve the welding toughness of the steel by pinning the austenite grain boundary and inhibiting the grain growth. For the evolution behaviors of different second phases, such as Ostwald ripening, dissolution and precipitation in welding thermal cycle greatly affect the microstructure and properties of HAZ in high strength steel. Thus, it is necessary to study the relationship between RE and the temperature, quantity, and size of the second phase precipitation in steel. Lin et al. [93] studied the effect of RE in Nb(Ti,V) microalloyed steel on the precipitation of second phases such as Nb(C,N), (Nb,Ti) (C,N), and V(C,N). The results indicated that RE reduced the complete dissolution temperature of the second phase, inhibited its precipitation in the austenite zone, and promoted its precipitation in the ferrite zone through increasing the incubation period and decreasing the precipitation rate. The research results of Zhou et al. [94] also showed that RE could increase the precipitation rate and quantity of V(C,N) in the ferrite zone. You et al. [95] studied the interaction between La and alloy elements in bcc-Fe from the perspective of the electron layer and believed that the mutual repulsion between La and Nb promoted the precipitation of NbC in the ferrite zone.

However, there are few reports on the accurate determination of RE in solid solution. The dissolution and precipitation behavior of Ti, Nb, and V in austenite and ferrite lack quantitative experimental measurement, and the corresponding thermodynamic and kinetic calculations should be systematically studied. In addition, whether the pinning effect of disperse high melting point RE inclusions on austenite grain boundary is consistent with that of the second phase in the welding process of high strength steel and their contributions need to be explored.

## 5. Conclusions

With the deepening of relevant research, the mechanism of oxide metallurgy technology on the weldability of high strength steel is increasingly clear, but there are still application difficulties and content to be further studied. The following conclusions are drawn by analyzing the application of oxide metallurgy technology and RE:

(1) Optimizing the alloy content in high strength steel and controlling the austenite grain size and cooling speed can help to improve the microstructure and properties of HAZ. Based on the principle of oxide metallurgy technology, the additions of Ti, Ca, Mg, and Zr can form fine and dispersed inclusion particles in steel, which effectively pin austenite grain boundary and induce AF nucleation, leading to enhancing the welding toughness of high strength steel.
(2) Adding an appropriate amount of RE into high strength steel-low carbon equivalent can purify liquid steel and modify inclusions. The disperse high melting point RE inclusions are also able to pin grain boundary and promote the nucleation of AF, and thus can improve the fracture toughness of HAZ. However, it is necessary to further study the effect of key trace elements on RE inclusions, the coordinated control of RE with O and S, the effective control of RE inclusion size, and the stable control of RE content in steel.
(3) The improvement of weldability of high strength steel with high carbon equivalent is still a technical difficulty in industrial production. To solve the weldability problem, RE and RE inclusions are able to improve the microstructure and properties of HAZ by acting on microstructure transformation, grain refinement, and pinning grain boundary. The mechanisms of dissolution, precipitation, and pinning grain boundary of the second phase, and the pinning mechanism of RE inclusions need to be further analyzed in RE containing steel.

## Figures and Tables

**Table 1:** Research on RE oxide metallurgical technology.

Carbon Content/%	Carbon Equivalent	Rare Earth Elements and Content/%	Rare Earth Inclusions	Microstructure	Author	Time
0.12	0.27	0.02 Ce	Ce_2_O_2_S + CeAlO_3_	BF, P, IAF	Wen et al.	2012 [61]
0.04	0.30	0.0014 (Ce-La)	Ti-RE-Zr complex oxide	AF	Nako et al.	2015 [62]
0.15	0.41	0.006 Ce + 0.003 La	(La-Ce-Ca-Al-Mg-S) complex oxide	PF, F	Chu et al.	2018 [63]
0.12	0.34	0.021 (Ce-La)	RE_2_O_2_S + MnS	AF, GBF	Song et al.	2019 [64]
0.05	0.36	0.05 Ce	CeAlO_3_	AF, B	Cao et al.	2019 [10]
0.17	0.40	0.02 La	La_2_O_2_S + MnS	AF, FSP, GBF	Xie et al.	2020 [65]

Abbreviations: BF, block ferrite; P, pearlite; IAF, intragranular acicular ferrite; AF, acicular ferrite; GBF, grain boundary ferrite; B, bainite; FSP, ferrite side plate.

**Table 2:** Effect of Ce on austenite grain size. (Reproduced with permission from [84]. Copyright 1994 China Academic Journal Electronic Publications.)

Solid Solubility of Ce/%	Grain Size at Different Temperatures (μm)			
	1073 K	1123 K	1173 K	1223 K
0	11.1	12.7	12.9	20.3
0.0097	8.6	9.5	10.8	12.5
0.0399	7.5	7.7	8.1	9.5
0.0977	6.2	6.4	6.5	6.7

## Data Availability

Not applicable.
